# FoxC2 Enhances BMP7-Mediated Anabolism in Nucleus Pulposus Cells of the Intervertebral Disc

**DOI:** 10.1371/journal.pone.0147764

**Published:** 2016-01-29

**Authors:** Zheng Wang, Changfeng Fu, Yong Chen, Feng Xu, Zhenyu Wang, Zhigang Qu, Yi Liu

**Affiliations:** 1 Department of Spinal Surgery,The First Hospital of Jilin University, No.71,Xinmin Avenue, Chaoyang District, Changchun, Jilin Province 130021,China; 2 Department of Neurosurgery, The First Hospital of Jilin University, No.71, Xinmin Avenue, Chaoyang District, Changchun, Jilin Province 130021, China; University of Crete, GREECE

## Abstract

Bone-morphogenetic protein-7 (BMP-7) is a growth factor that plays a major role in mediating anabolism and anti-catabolism of the intervertebral disc matrix and cell homeostasis. In osteoblasts, Forkhead box protein C2 (FoxC2) is a downstream target of BMPs and promotes cell proliferation and differentiation. However, the role FoxC2 may play in degenerative human intervertebral disc tissue and the relationship between FoxC2 and BMP-7 in nucleus pulposus (NP) cells remain to be elucidated. This study aims to investigate the presence and signaling mechanisms of FoxC2 in degenerative human intervertebral disc tissue and NP cells. Western blot and real-time quantitative reverse transcription polymerase chain reaction (qRT-PCR) analyses were used to measure FoxC2 expression in the NP tissue and cells. Transfections were carried out to measure the effect of FoxC2 on BMP-7-mediated extracellular matrix upregulation. Adenoviral knock-down of Smad1 was performed to investigate the mechanism of BMP-7-induced FoxC2 expression. In degenerative NP tissue, FoxC2 was markedly upregulated and positively correlated with increased disc degeneration. Induction of NP cell proliferation was confirmed by using cell counting kit-8 assay, immunocytochemistry and real-time qRT-PCR for Ki67. FoxC2 led to decreased noggin expression and increased Smad1/5/8 phosphorylation. During combined treatment with BMP-7, FoxC2 greatly potentiated anabolism through synergistic mechanisms on ECM formation. Combination therapy using BMP-7 and FoxC2 may be beneficial to the treatment of intervertebral disc degeneration.

## Introduction

Degenerative disc disease (DDD) is thought to be a major cause of lower back pain which interferes with normal activities and impacts the ability to work [1]. Although the process of DDD remains unclear, progressive breakdown of the extracellular matrix (ECM) is thought to be one of the most important causative factors. Changes in the structure of the ECM are associated with adverse genetic, biomechanical and nutritional factors [2]. In the course of DDD, the breakdown of the ECM from age changes and adverse biomechanical loading can be enhanced by a genetically inferior matrix [3]. The interplay among diversified cytokines, growth factors, matrix-degrading enzymes and their inhibitors forms an intricate balance between anabolic and catabolic processes [4, 5]. Among these, matrix metalloproteases (MMPs) and aggrecanases increase expression of matrix-degrading enzymes, and both are induced by various proinflammatory cytokines [6–9]. Hiyama *et al*. found activation of WNT/β-catenin signaling promotes the breakdown of the matrix and may modulate MMP and TGFβ signaling in NP cells [10]. In addition, previous reports suggest that TGF-β signaling pathway is important in the regulation of degenerative processes in IVDs [11, 12]. In degenerative discs, aggrecan and collagen II are observed to be most notably downregulated in the ECM [13]. To ameliorate progression of disc degeneration, growth factors have been used to shift the metabolic status from catabolic to anabolic to reestablish their balance [14]. Further understanding of the pathophysiological mechanisms of DDD may help verify biological treatments, capable of promoting ECM repair and regeneration [15].

Bone-morphogenetic proteins (BMPs), which constitute the largest subgroup of the transforming growth factor-β (TGF-β) superfamily, are often used as therapeutic factors in treating disc degeneration [16, 17]. BMP-7 is expressed in NP cells whereby it exerts potent effects on cell anabolism and differentiation [18]. BMP-7 can stimulate the production and formation of aggrecan and Collagen II via the Smad1/5/8 signaling pathways [19]. Recently, Zhang *et al*. found BMP-7 has a similar anabolic effect on ECM metabolism in bovine intervertebral disc (IVD) cells [20]. Furthermore, similar findings are reported by Masuda and Imai in rabbit or human IVD cells, providing further evidences for the functional role BMP-7 plays in decreasing intervertebral disc (IVD) degeneration[21, 22]. Takegami *et al*. report that after ECM depletion in response to IL-1, BMP-7 can effectively increase the expression of IVD ECM genes by rabbit NP cells[23]. These results demonstrate the importance of BMP-7 signaling pathways during the occurrence and development of DDD, and support using BMP-7 for the treatment of DDD is a feasible biological approach.

FoxC2 is a member of the forkhead box (FOX) family of transcription factors. When the abbreviation of FOXC2 contains all uppercase letters, this refers to human form. When Foxc2 has only the first letter capitalized, this refers to mouse, and when FoxC2 has the first and subclass letters capitalized, this refers to all chordates [24]. Foxc2 has been shown to be involved in cancer metastases, epithelial-mesenchymal transition (EMT) and lymphedema-distichiasis syndrome [25, 26].

Recently, Nifuji *et al*. have shown that following treatment of skeletal precursors with BMPs, Foxc2 may play a role in the early stage of chondrogenic differentiation, because increased expression of Foxc2 is observed in relation to SOX9, noggin and aggrecan [27]. Furthermore, Park *et al*. have found that Foxc2 is a downstream target of BMP-2 and promotes the survival, proliferation and differentiation of osteoblasts [28]. Recent studies imply that BMP signaling is required for Foxc2 expression and that Foxc2 acts as a modulator of the pathway [29]. A similar study has found that forced expression of FOXC2 in C2C12 cells results in upregulation of BMP-4 [30]. Overall, these results suggest that Foxc2 is a vital element of ECM secretion via facilitation of BMP signaling pathways and failure of proper Foxc2 transcript regulation may result in DDD. BMPs play an important role in regulating anabolic processes of the NP cells in which FoxC2 may have significant effect in DDD pathogenesis. The key question we address here is to demonstrate the relationship between FoxC2 and degenerative human intervertebral disc, as well as the functional role of FoxC2 on the proliferation and anabolism of primary cultured rat NP cells. Specifically, we also elucidate the underlying molecular mechanism between FoxC2 and BMP signaling pathways.

## Materials and Methods

### Ethics statement

The study protocol was approved by the Institutional Ethics Committee of the First Hospital of Jilin University. Human lumbar and cervical IVD samples were obtained from patients undergoing spinal surgeries during March 5^th^, 2014 to March 8^th^,2015, following approval from the Institutional Review Board of the First Hospital of Jilin University. Patients consented to the collection and use of tissue samples for research and provided written informed consent in all cases. All the human tissue samples used for this study were obtained from patients as a standard part of the patients' spinal surgeries without additional biopsies. All animal experiments were carried out in a humane manner after receiving approval from the Institutional Animal Experiment Committee of Jilin University and in accordance with the Regulation for Animal Experiments and Fundamental Guideline for Proper Conduct of Animal Experiments of our university.

### Reagents

BMP-7 inhibitor noggin was purchased from Protect (Rocky Hill, NJ, USA). All other chemicals and reagents were purchased from Sigma (Oakville, ON, Canada) unless otherwise specified.

### Patients and samples

Patient samples were collected and isolated as previously described by Yu *et al*[31]. We collected human lumbar NP specimens from 36 patients with lumbar disc degeneration (average age 47.36±6.651, range 33–57 years) and 4 patients with idiopathic scoliosis as a control (average age 19.75±1.258, range 18–21 years). All these patients had taken routine magnetic resonance imaging (MRI) scans of the lumbar spine before the operation. A modified Pfirrmann classification was used to grade the degree of disc degeneration from T2-weighted images. According to the modified classification system of the International Society for the Study of the Lumbar Spine [32], 7 of these patients were sequestration (S), 10 were transligamentous extrusion (TE), 8 were subligamentous extrusion (SE) and 11 were protrusions (p). Four of the 36 samples were obtained from the level of L3–L4, 17 from L4–L5, and 14 from L5–S1. After excluding the herniation and granulation tissue, we washed these tissue specimens three times with phosphate-buffered saline (PBS) and separated NP from the annulus fibrosus (AF) using a stereotaxic microscope and finally frozen them in liquid nitrogen (Table 1).

**Table 1:** Clinical Finding in 50 Patients with LDH.

Patient No.	Sex	Age(yr)	Level	Type of LDH	MRI Scores
1	F	48	L5/S1	TE	4
2	F	33	L4/L5	TE	2
3	M	43	L5/S1	S	5
4	F	51	L4/L5	SE	4
5	F	39	L4/L5	TE	3
6	M	45	L5/S1	P	4
7	F	55	L4/L5	SE	5
8	M	52	L5/S1	S	5
9	M	43	L4/L5	P	4
10	M	38	L4/L5	SE	2
11	F	53	L4/L5	SE	5
12	M	47	L3/L4	P	4
13	F	41	L5/S1	P	3
14	F	52	L5/S1	SE	4
15	F	49	L4/L5	TE	5
16	F	55	L3/L4	SE	4
17	M	39	L4/L5	P	3
18	M	51	L4/L5	S	5
19	F	53	L5/S1	S	4
20	M	51	L5/S1	TE	4
21	M	43	L4/L5	P	3
22	M	48	L5/S1	TE	5
23	F	56	L5/S1	TE	3
24	F	36	L3/L4	P	4
25	M	57	L4/L5	TE	4
26	M	46	L5/S1	P	5
27	F	52	L3/L4	S	5
28	M	54	L4/L5	TE	3
29	M	43	L5/S1	P	5
30	M	51	L4/L5	SE	5
31	M	53	L5/S1	S	4
32	F	46	L5/S1	P	3
33	F	42	L4/L5	S	5
34	F	52	L4/L5	TE	4
35	M	55	L4/L5	SE	3
36	F	33	L4/L5	P	3

LDH, lumbar disc herniation; F, indicates female; M, male; P, protrusion; S, sequestration; SE, subligamentous extrusion; TE, transligamentous extrusion; L, lumbar; S,sacral; MRI, magnetic resonance imaging

### Rat NP cells isolation and culture

Disc tissue was harvested from male Wistar rats (200–250 g). All the rats were purchased from Animal Experimental Center of Jilin University and were killed with a lethal dose of 10% chloral hydrate. Lumbar and tail IVDs were separated from the spinal column under aseptic conditions. After separation of the NP, the tissue was cut into 1-mm^3^ blocks, which were treated separately with 0.1% collagenase II for 4‒6 hours [33]. NP cells were released by enzymatic digestion in Dulbecco’s modified Eagle’s medium (DMEM) and 10% fetal bovine serum (FBS) supplemented with 50 U/mL penicillin and 50 μg/mL streptomycin in a humidified atmosphere containing 5% CO_2_ at 37°C. Cells were then passaged and further cultured in the presence of BMP-7 (10‒100 ng/ml). On reaching approximately 80% confluence, cells were subcultured in 60-mm culture dishes (4×10^5^ cells/well). All experiments in this study used first- or second-generation cells.

### Cell transfection

According to the manufacturer’s instructions, Lenti-FoxC2, Lenti-GFP or scramble was transfected into the cells by DharmaFECT1 Reagent (Dharmacon, TX, USA) at a final oligonucleotide concentration of 10 nmol/L. For each cell transfection, two replication experiments were performed.

### Western blot analysis

Cell and tissue lysates were prepared using modified radioimmunoprecipitation assay (RIPA) buffer as previously described [34]. Total protein concentrations of cell lysates were determined using the bicinchoninic acid (BCA) protein assay (Pierce, Rockford, IL, USA). Equal amounts of protein (35 μg per lane) were resolved by 10% SDS-PAGE and transferred to a nitrocellulose membrane for western blot analyses as described previously [35]. Immunoreactivity was visualized using the ECL system (Amersham Biosciences, Piscataway, NJ, USA).

### Real-time PCR

PCR amplification was carried out in accordance with the previously described method. Total RNA was isolated using TRIzol reagent (Invitrogen, Carlsbad, CA) following the manufacturer’s instructions. cDNA was then reverse transcribed according to the manufacturer’s instructions. Quantitative PCR was carried out using a SYBR green Jumpstart Taq ReadyMix (TaKaRa) on a Roche LightCycler 480. A threshold cycle for each PCR amplification was subjected to 35 cycles of 95°C for 15 s and 54°C for 2 min. The primer sequences and their conditions for use were summarized in Table 2. Glyceraldehyde 3-phosphate dehydrogenase (GAPDH) was used to normalize expression levels.

**Table 2:** Primer Sequences for PCR.

Primers	Forward	Reverse	Tm (°C)
FoxC2	5’-CGCCTAAGGACCTGGTGAAG-3’	5’-GGAAGCGGTCCATGATGA-3	56
BMP-7	5’-GGTGGCAGGACTGGATCATC-3’	5’-GCGCATTCTCCTTCACAGTAATAC-3’	56
Aggrecan	5’-AAGGACTGTCTATCTGCACGCCAA-3’	5’-TCACCACCCACTCCGAAGAAGTTT-3’	56
Collagen Type II	5’-TCATAAGGATGTGTGGAAGCCCGA-3’	5’-GGCTGAGGCAGTCTTTCATGTCTT-3’	56
Noggin	5’-AGCGAGATCAAAGCGCTGGAGTT-3’	5’-TCTGTAACTTCCTCCGCAGCTTCT-3’	56
Ki-67	5’-TCCTTTGGTGGGCACCTAAGACCTG-3	5’-TGATGGTTGAGGTCGTTCCTTGATG-3	56

### Immunofluorescence microscopy

Cells were plated in flat bottom 96-well plates (4×10^3^/ well) and treated with BMP-7 for 1‒24 h. After incubation, cells were fixed with 4% paraformaldehyde, permeabilized with 0.2% triton-X 100 in PBS for 10 min, blocked with PBS containing 5% FBS, and incubated with antibodies against FoxC2 (1:200), aggrecan (1:100), SOX9 (1:200), and MMP-2 (1:200) at 4°C overnight. As a negative control, cells were reacted with isotype IgG under similar conditions. After washing, the cells were incubated with DyLightTM488-conjugated goat anti-rabbit antibody and fluorescein- or DyLightTM594-conjugated goat anti-mouse antibody (Jackson Laboratory), at a dilution of 1:200 for 45 min at room temperature. Fluorescence signals were captured using an Olympus Fluoview FV1000 confocal microscope and analyzed by the FV10-ASW 1.6 Viewer program (Olympus, Japan).

### Cell Counting Kit-8 (CCK8) assay

NP cells were seeded in 96-well plates at a density of 1,000 cells per well with 100 μl of DMEM/F12 medium. Cells were then cultured for another 0, 24, 48, 72 or 96 h. The supernatants were removed and 100 μl of DMEM/FBS medium containing 10 μl of CCK-8 was added to each well prior to incubating for 3 h at 37°C. The culture plates were then shaken for 10 min and the optical density (OD) values were read at 450 nm.

### Statistical analysis

All measurements were performed in triplicate and data were presented as means ± standard deviation (SD). Differences between groups were analyzed by Student’s t test; *p<0.05, ** p<0.01.

## Results

### Expression of FOXC2 in degenerative NP tissue and correlation with degeneration grade

**Fig 1:**
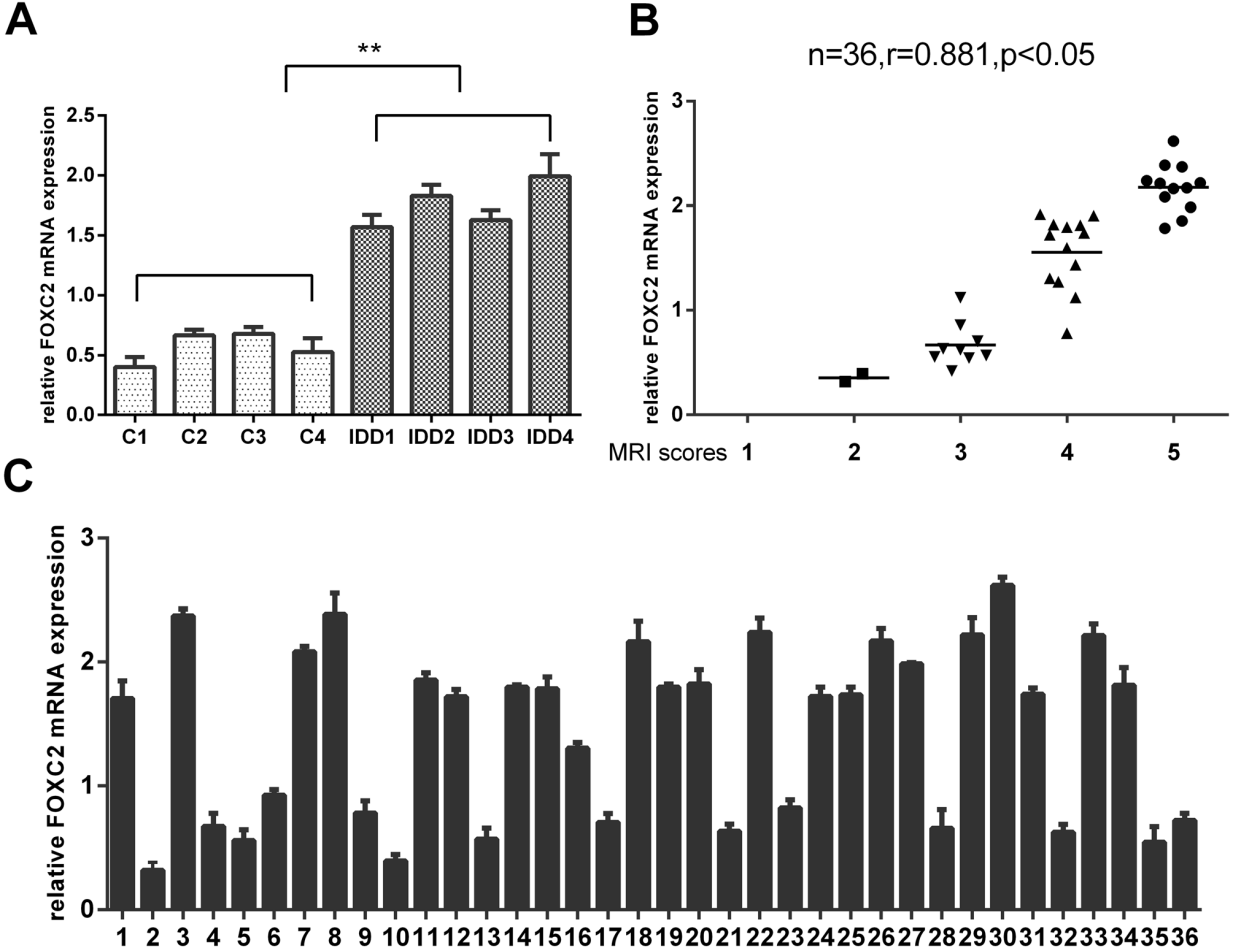
The expression of FOXC2 in human nucleus pulposus tissue. (**A**) The expression of FOXC2 exhibited significantly higher levels in 4 degenerative nucleus pulposus tissue compared to 4 idiopathic scoliosis nucleus pulposus tissue. (**B**) The correlation between the expression of FOXC2 and Pfirrmann scores of disc degeneration grade. n = 36,r = 0.881,p<0.05. (**C**) RT-PCR analysis of FOXC2 in the human nucleus pulposus tissue of 36 patients. Error bars represent SD. As compared with control, ** indicates p<0.01.

Of the 36 DDD patients, 19 females and 17 males, the average age of these patients was 47.36±6.651 (range 33–57 years). The clinical findings for these patients were summarized in Table 1. FOXC2 expression in the degenerative NP tissue was significantly higher compared to the idiopathic scoliosis NP tissue as determined by real-time PCR (Fig 1A). With respect to the expression of FOXC2 in the 36 DDD patients (Fig 1B and 1C), no significant difference was observed between samples from different herniation types or genders. However, expression of FOXC2 was positively correlated with the disc degeneration grade (n = 36, r = 0.881, p<0.05).

### Immunofluorescence and Real-Time PCR validation of primary cultured rat NP cells

**Fig 2:**
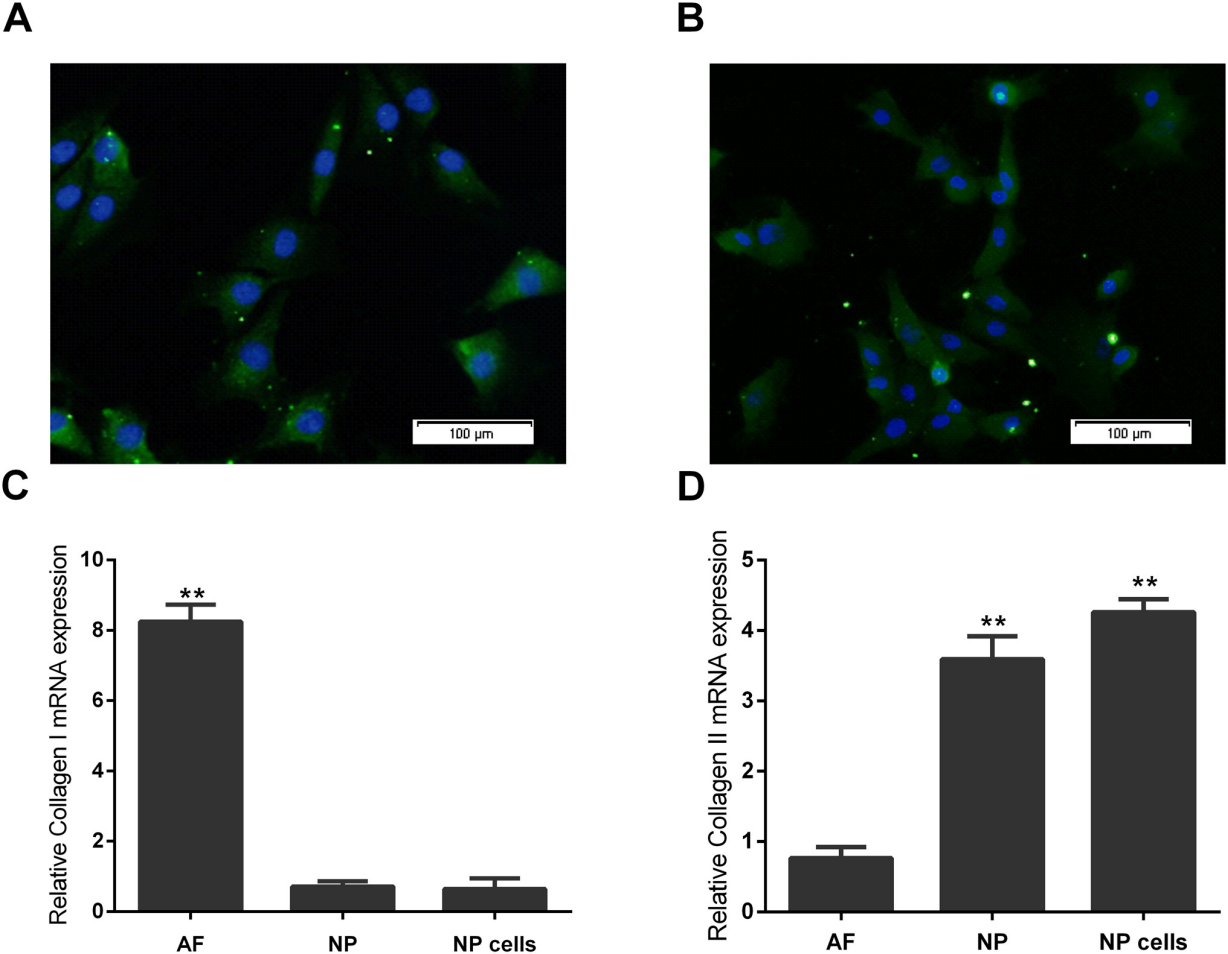
Immunofluorescence characterization and real-time quantitative PCR validation of primary cultured human NP cells. (**A**) Fluorescence microscopy images showing collagenase type II was observed in NP cells. Nuclei were stained with DAPI, shown in blue. Images were acquired using laser scanning confocal microscopy under a 40×objective. (**B**) Fluorescence microscopy images showing aggrecan was observed in NP cells. Nuclei were stained with DAPI, shown in blue. Images were acquired using laser scanning confocal microscopy under a 40×objective. (**C**) Real-time RT-PCR analysis of negative NP cell marker gene collagen type I in NP cells, NP and AF. Real-time RT-PCR analysis was performed in triplicate and the expression levels of collagenase type I mRNAs were normalized to GAPDH mRNAs. (**D**) Real-time RT-PCR analysis of NP cell marker gene collagen type II in NP cells, NP and AF. Real-time RT-PCR analysis was performed in triplicate and the expression levels of collagenase type II mRNAs were normalized to GAPDH mRNAs. Error bars represent SD. As compared with control, ** indicates p<0.01.

After approximately 7‒10 days, the Primary NP cells reached complete confluence. The cells were round or polygonal with a granular cytoplasm. Collagen II and aggrecan expression in these cells were observed by immunofluorescence (Fig 2A and 2B). As earlier studies indicated, collagen type I had significantly lower expression levels in NP cells and NP tissue than in AF tissue (Fig 2C), whilst Collagen II was found to have significantly higher expression levels in NP cells and NP tissue than in AF tissue (Fig 2D).

### FoxC2 induced NP cells proliferation

**Fig 3:**
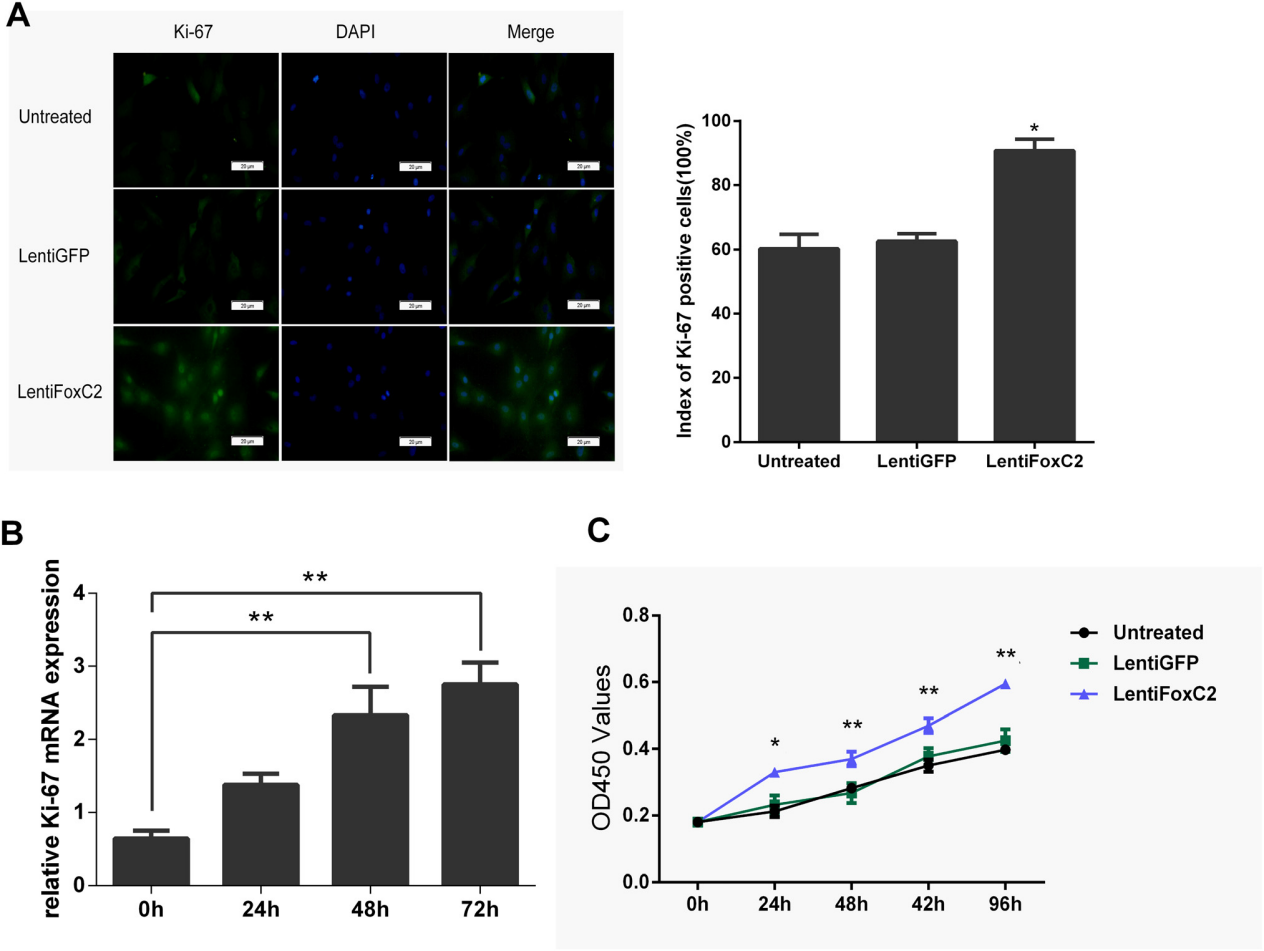
Overexpression of FoxC2 promotes NP cells growth. (**A**) Immunohistochemical staining of NP cells against Ki-67. Nuclei were stained with DAPI, shown in blue. Images were acquired using laser scanning confocal microscopy under a 40×objective, and had Ki-67-positive percentages in cultured NP cells 48h after transfection with LentiFoxC2 or LentiGFP or no transfection. (**B**) Real-time RT-PCR analysis of Ki-67 mRNA expression in NP cells after transfection of LentiFoxC2 for 0h, 24h, 48h or 72h. (**C**) Growth of NP cells was shown after transfection with 50 nmol/L of LentiFoxC2 or LentiGFP or no transfection. The growth index was assessed at 1, 2, 3, 4, and 5 days. Error bars represent SD. As compared with control, * indicates p<0.05, ** indicates p<0.01.

To determine whether up-regulation of FoxC2 in NP cells induce survival, proliferation and differentiation of NP cells, we transfected Lenti-FoxC2 in NP cells and transfected Lenti-GFP as the control. Transfecting Lenti-FoxC2 in NP cells significantly increased the expression of Ki-67, a famous proliferative marker, in immunofluorescence staining (Fig 3A). Compared with the control or untreated groups, the percentage of Ki-67-positive NP cells was obviously increased in the group transfected with Lenti-FoxC2. Also, transfecting Lenti-FoxC2 in NP cells induced the up-regulation of Ki-67 mRNA level as a time-dependent manner, and the Ki-67 mRNA level was significantly increased after 48h and 72h when compared to control (Fig 3B). We used CCK-8 proliferation assay to compare the effects of Proliferation between Lenti-FoxC2 and Lenti-GFP transfected to NP cells. As shown in Fig 3C there was a significant increase of cell proliferation in the Lenti-FoxC2 transfected NP cells compared with the control.

### BMP-7 induces FoxC2 expression in NP cells

**Fig 4:**
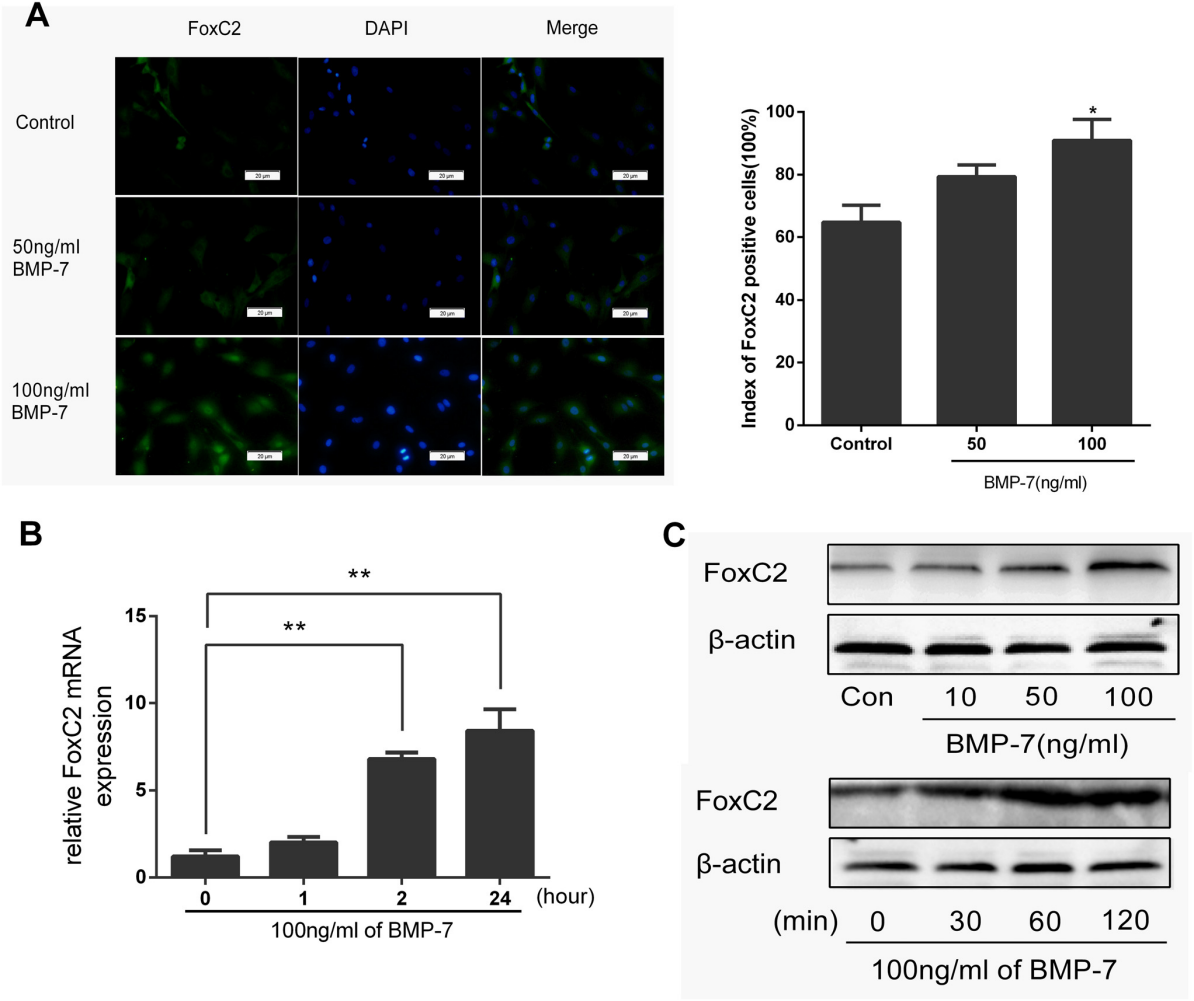
Immunohistochemical staining of NP cells against FoxC2. (**A**) After 1-day serum deprivation, NP cells were treated 50 ng/ml or 100 ng/ml BMP-7 in serum-free medium for 2h. NP cells were Immunohistochemical stained against FoxC2. Nuclei were stained with DAPI, shown in blue. Images were acquired using laser scanning confocal microscopy under a 40×objective. FoxC2-positive percentages in cultured NP cells 2h after treated with 50 ng/ml or 100 ng/ml BMP-7 or control was under analysis. (**B**) NP cells were stimulated by BMP-7 (100 ng/ml) for the indicated periods of time. And then expression level of FoxC2 was measured by real-time RT-PCR. (**C**) NP cells were stimulated by 10, 50, and 100 ng/ml BMP-7 for 2h or NP cells were stimulated by BMP-7 (100 ng/ml) for the indicated periods of time. Cell lysates were then prepared and analyzed by western blotting with specific anti-FoxC2 antibody. β-actin was used as a control for normalization. Error bars represent SD. As compared with control, * indicates p<0.05, ** indicates p<0.01.

As mentioned above, BMPs likely played an important role during the process of FoxC2 expression [25]. We investigated whether BMP-7 could induce FoxC2 expression in NP cells. Immunofluorescence analysis confirmed this result, showing that BMP-7 treatment (50‒100 ng/ml, 2 h) promoted the expression of FoxC2 protein (Fig 4A), and higher concentrations of BMP-7 had a more significant effect compared with lower concentrations and the control. FoxC2 protein was induced by adding BMP-7 to the culture medium in a dose-dependent and time-dependent manner. The real-time qPCR results demonstrated that stimulation of rat NP cells cultured in monolayer with BMP-7 (100 ng/ml) induced a prominent increase after 2 hours in FoxC2 gene expression (Fig 4B). Western blots over the 60-min time frame showed that FoxC2 was significantly up-regulated at the optimal concentration of 100 ng/ml BMP-7 (Fig 4C).

**Fig 5:**
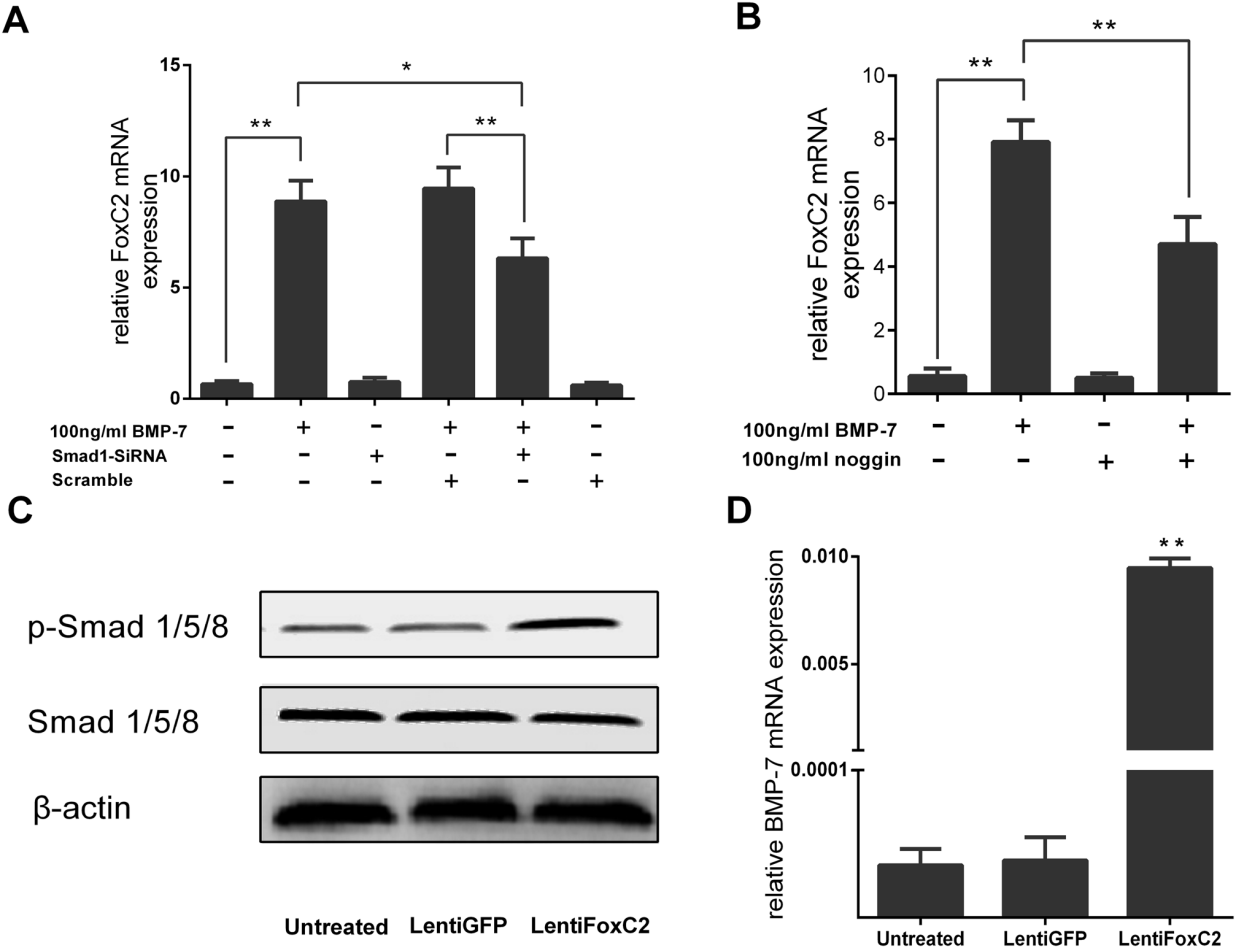
The interactional promotion effect between FoxC2 and BMP-7 signaling pathway. (**A**) FoxC2 mRNA expression levels were examined by real-time PCR in the presence and absence of SiRNA-Smad1 the BMP inhibitor noggin and Scramble. (**B**) NP cells were treated 100 ng/ml BMP7, 100 ng/ml noggin for 2h and the total RNA was extracted to perform real-time PCR of FoxC2 genes (**C**) Smad1/5/8 phosphorylation Protein expression in NP cells was transfected with 50 nmol/L of Lenti FoxC2 or Lenti GFP or no transfection. (**D**) Expression levels of BMP-7 were examined by real-time PCR after transfection of 50 nmol/L of Lenti FoxC2 or Lenti GFP or no transfection. Error bars represent SD. As compared with control, * indicates p<0.05, ** indicates p<0.01.

To determine whether increased FoxC2 by BMP-7 is related to the BMP signaling pathway, we quantified FoxC2 mRNA expression in the presence and absence of BMP-7, SiRNA-Smad1, Scramble and the BMP inhibitor noggin (Fig 5A and 5B). Concordant with our hypothesis, when the BMP signaling pathway was inhibited by SiRNA-smad1, this led to a significant downregulation of the anabolism effect that FoxC2 increased in NP cells, compared to in the presence of BMP-7 and Scramble or only in the presence of BMP-7. Also, the upregulation of FoxC2 by BMP-7 was significantly inhibited by noggin. Furthermore, we found that FoxC2 overexpression induced the phosphorylation of Smad/1/5/8 obviously, which were known to be activated by BMPs (Fig 5C). To further understand the relationship between FoxC2 and BMP-7, we measured mRNA levels of BMP-7 following FoxC2 overexpression. Under this condition, BMP-7 was positively upregulated whilst noggin was suppressed by FoxC2 (Fig 5D).

### FoxC2 enhances the matrix gormation dynergistic effect from the BMP-7 in NP Cells

**Fig 6:**
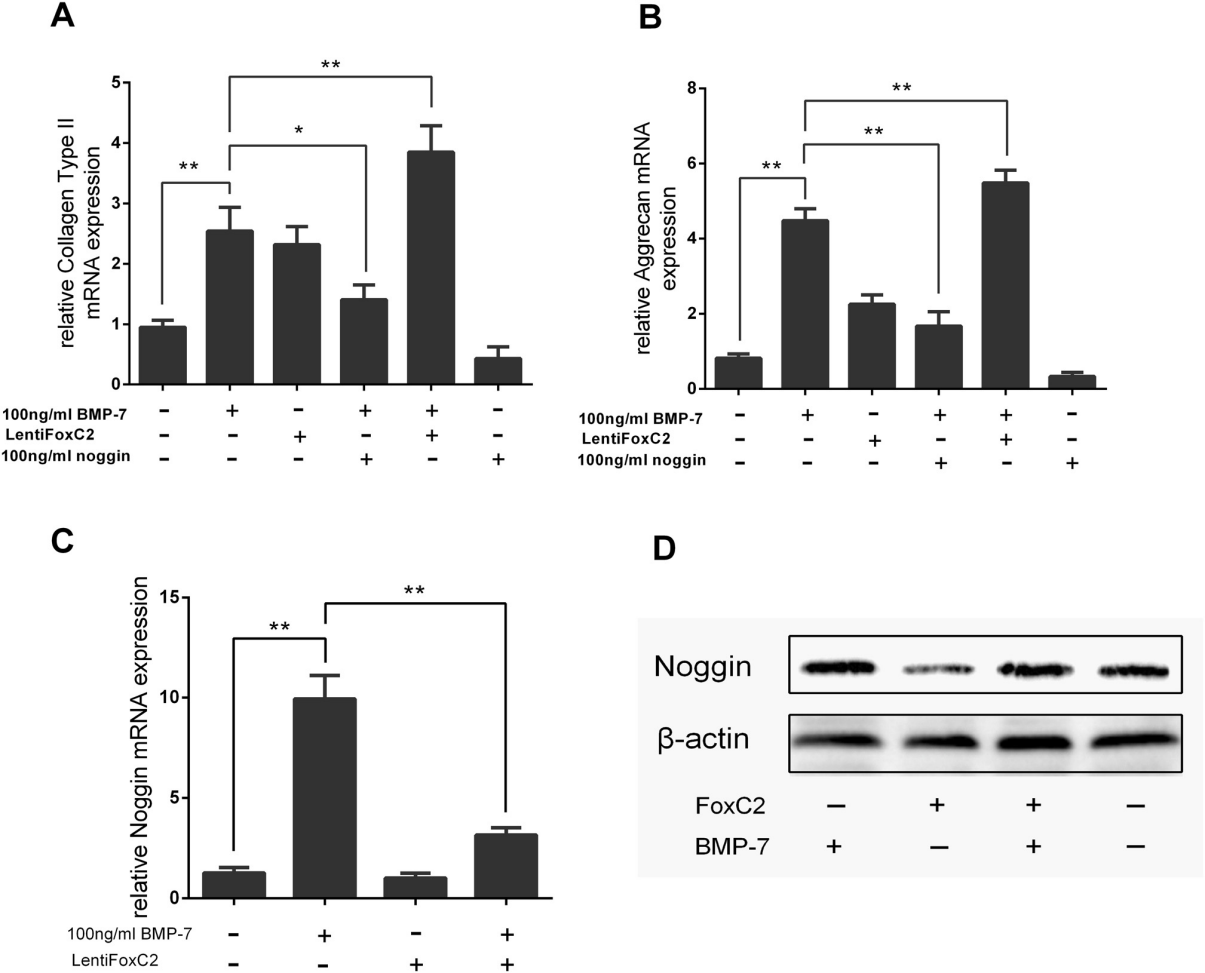
FoxC2 enhances the compensatory anabolic gene expression effect by BMP-7. NP cells were treated 100 ng/ml BMP7, 100 ng/ml noggin or 50 nmol/L of LentiFoxC2 for 2h. After stimulation, cells were harvested and the total RNA was extracted to perform real-time PCR of (**A**) collagen type-II genes and (**B**) aggrecan. (C) NP cells were stimulated by 50 nmol/L of Lenti FoxC2, BMP-7 (100 ng/ml), and cocktail of Lenti FoxC2 and BMP-7 for 2h. After stimulation, cells were harvested and the total RNA of Noggin was extracted to perform real-time PCR. As compared with control, ***p<0.01, *p<0.05, and **p<0.01. (**D**) Cell lysates were then prepared and analyzed by western blotting with specific anti-Noggin antibody. β-actin was used as the control for normalization. Error bars represent SD. As compared with control, * indicates p<0.05, ** indicates p<0.01.

We next examined the effects of FoxC2 on the expression of anabolic genes. As expected, the real-time qPCR results demonstrated BMP-7 stimulated the expression of ECM genes (Fig 6A and 6B). Specifically, FoxC2 had complementary effects on Collagen II and aggrecan expression, with FoxC2 respectively inducing a 2.4-fold (p<0.05) and 2.8-fold (p<0.01) increase in Collagen II and aggrecan gene expression when compared with controls. Moreover, the combination of FoxC2 with BMP-7 further increased Collagen II and aggrecan gene expression above levels induced by BMP-7 alone. In addition, we found that FoxC2 was effective against increased levels of noggin expression induced by BMP-7 (Fig 6C and 6D). These results suggest that FoxC2 enhances BMP7-mediated upregulation of anabolic genes. Taking together, we propose that FoxC2 blocks the positive feedback regulation of BMP-7 by noggin in NP cells, likely through the crosstalk between other signaling pathways.

## Discussion

Disc degeneration is a complex process caused when the delicate balance between anabolic and catabolic processes is broken. From previous researches at the molecular level, we know that genes involved in NP cell degeneration are tightly regulated and coordinated by several signaling pathways [10, 36–38]. It is known that activation of the transcription factor Smad through the BMP/Smad signaling pathways is essential for enhance ECM repair and regeneration in NP cells [39, 40]. However, during disc degeneration, the molecular mechanisms for the regulation and coordination between these pathways remain unclear. In previous studies, FoxC2 is reported to serve as a key regulator in diverse biological and pathological processes, including cell proliferation, differentiation and apoptosis [41–43]. However, whether FoxC2 has pathogenic significance in degenerative NP tissue and the significance of FoxC2 during the process of DDD are yet to be elucidated. In this study, we provide evidence for a novel mechanism whereby FoxC2 plays an important role in promoting NP cell proliferation and stimulates synthesis of the ECM by regulating the BMP signaling pathways.

We demonstrated that FoxC2 was frequently upregulated in human degenerative NP tissue and significantly associated with disc degeneration grade. We cultured primary NP cells from rat discs and characterized them by assessing cell morphology and expression of Collagen I, Collagen II and aggrecan. The characteristics of NP cells in our study were consistent with those of previous studies [30, 44]. The CCK-8 assay showed that overexpression of FoxC2 increased the NP cell proliferation. Moreover, immunostaining and Real-time RT-PCR analysis for the proliferation marker Ki-67 with or without FoxC2 offered further reassurance of these findings. In this regard, FoxC2 expression in human NP tissue, as well as in other tissue, may play a similar vital regulatory role in cell proliferation and differentiation. Overexpression of FoxC2 induced increased NP cell proliferation, suggesting that FoxC2 may be involved in mechanisms of the development process of the NP cells. It should be noted that FoxC2 could regulate cell proliferation via several signaling pathways, therefore more comprehensive profiling should be carried out. Further experiments are also required to pinpoint the effects of FoxC2 on cell death and specific phases of the cell cycle.

BMP signals are mediated by type I and II BMP receptors and their downstream molecules Smad1, 5 and 8. Thereafter, when type I receptor kinase is activated, Smad1, 5 and 8 proteins are phosphorylated and form a complex with Smad4 to translocate into the nucleus. Within the nucleus, they then interact with other transcription factors and regulate expression of target genes. Together with other transcription factors and transcription coupling factors, the Smad complexes bind to Smad binding elements (SBEs) in the promoters of target genes whereby they regulate expression of these genes[16]. The anabolic effects of BMP-7 have been shown through the stimulation of matrix biosynthesis in NP cells [22]. Although previous studies have indicated BMPs regulates FoxC2 expression [25], the exact role of FoxC2 in the BMP signaling pathway is not fully understood. Consistent with other types of cells, BMP-7 also can induce FoxC2 expression in NP cells [45]. We clarified the optimal concentration and time required for BMP-7 to significantly upregulate FoxC2 expression. When the BMP-7 signaling pathway was inhibited by SiRNA-Smad1 or noggin, expression of FoxC2 was reduced significantly. The regulation of FoxC2 by BMP-7 in NP cells was further corroborated by immunofluorescence analysis in which FoxC2 levels were observed to be much higher in NP cells after addition of extrinsic BMP-7 when compared with the controls. These results indicated that FoxC2 expression was associated with DDD, and BMP-7 regulated FoxC2 expression in NP cells.

To further understand the relationship between FoxC2 and BMP pathways, we demonstrated that overexpression of FoxC2 activated a positive regulation mechanism. Noggin was known to inhibit the BMP pathways in a negative feedback system. Overexpression of FoxC2 was found to efficiently downregulate noggin expression, thereby indirectly reducing the inhibition effect of noggin within the BMP pathways. Furthermore, overexpression of FoxC2 resulted in phosphorylation of BMP7-responsive SMAD proteins (Smad1/5/8), that modulated BMP7-SMAD1/5/8-dependent anabolic target genes in NP cells. We therefore speculated that FoxC2 played a crucial role in sustaining activation of the BMP pathways.

Results from previous studies demonstrated that BMP-7 had the ability to stimulate aggrecan and collagen synthesis in human disc cells. In this study, further evidence demonstrated that both FoxC2 and BMP-7 had potent anabolic effects on rat NP cells. We found that FoxC2 enhanced the upregulation effect of BMP-7 for ECM proteins like Collagen II and aggrecan. These results not only supported our previous conclusion about the FoxC2 positive regulation effect in BMP signaling, but also provided evidence for the benefits of a novel combination treatment in disc degeneration.

Several studies have previously shown that FoxC2 can induce the BMP pathway through crosstalk with other signaling pathways. Fujita *et al*. demonstrated that Foxc2 was a common transcriptional activator of TGF-signaling [42]. Although BMP is a subgroup of the TGF-β superfamily, they both have an anabolism effect in NP cells. In addition, BMP and TGF-β pathways downstream of Smad transcription factors are different but both require Smad4 for their activation, therefore a competitive relationship exists between BMP and TGF-β pathways [46]. Gozo *et al*. have shown that forced expression of FOXC2 promoted upregulation of Wnt4 expression and activation of increased levels of BMP [30]. It is well-known that activation of WNT/β-catenin signaling modulates MMPs and TGFβ/BMP signaling in NP cells, increasing breakdown of the matrix and thereby promoting IVD degeneration [10, 47]. This might partially explain why FoxC2 induced by BMP-7 has a positive feedback effect to BMP pathway. Further studies are required to determine the exact molecular mechanisms by which FoxC2 acts as a modulator between the Wnt, BMP and TGF-β signaling pathways in NP cells.

**Fig 7:**
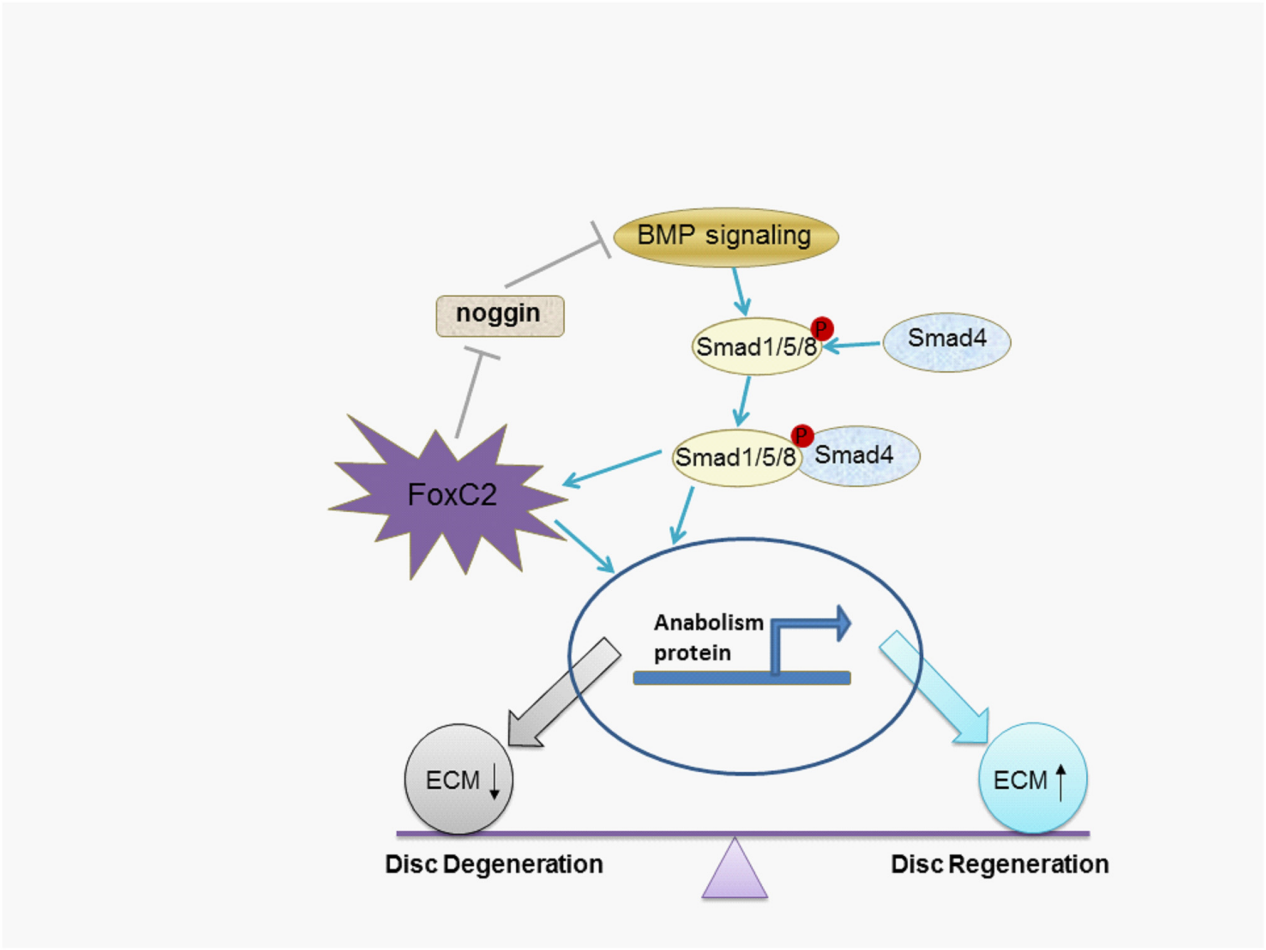
Schematic diagram of FoxC2-mediated biological effects on the BMP7-SMAD signaling pathways. BMP/Smad signaling pathway can upregulate FoxC2 expression in the NP cells. **FoxC2** inhibits the production of noggin and enhances BMP7-mediated anabolism, what was a positive feedback for maintaining signal activation.

In conclusion, we identified that the transcription factor FoxC2 was over expressed in human degenerative discs. The mRNA expression level of FoxC2 was positively correlated with disc degeneration grade. Furthermore, we demonstrated that overexpression of FoxC2 increased the proliferation of NP cells. Importantly, BMP-7 induced FoxC2 expression, which was a positive feedback for maintaining signal activation (Fig 7). Our findings suggest that upregulation of FoxC2 results in increased anabolic effects. Future studies are warranted to elucidate the relationship between FoxC2 and other signaling pathways and crosstalk between them.
